# Superior Ionic Transferring Polymer with Silicon Dioxide Composite Membrane via Phase Inversion Method Designed for High Performance Sodium-Ion Battery

**DOI:** 10.3390/polym12020405

**Published:** 2020-02-11

**Authors:** Ponnaiah Arjunan, Mathiyalagan Kouthaman, Rengapillai Subadevi, Karuppiah Diwakar, Wei-Ren Liu, Chia-Hung Huang, Marimuthu Sivakumar

**Affiliations:** 1 Department of Physics, #120, Energy Materials Lab, Science Block, Alagappa University, Karaikudi 630 003, Tamil Nadu, India; nano.arjun@gmail.com (P.A.); kouthaman1993@gmail.com (M.K.); selfindicator@gmail.com (K.D.); 2 Department of Chemical Engineering, R&D Center for Membrane Technology, Research Center for Circular Economy, Chung-Yuan Christian University, Chung-Li 32023, Taiwan; wrliu@cycu.edu.tw; 3 Development Centre, Metal Industries Research, Kaohsiung 81160, Taiwan; chiahung@mail.mirdc.org.tw

**Keywords:** PVdF-SiO_2_ membrane, phase inversion method, superior ionic conductivity, high capacity delivery

## Abstract

Superior sodium-ion-conducting polymer poly(vinyledene fluoride)–silicon dioxide (PVdF-SiO_2_) composite separator membrane was prepared via simple phase inversion method, which is a suitable alternative conventional polypropylene membrane. Basically, PVdF is the promising for use as high porous polymer electrolyte membrane due to its high dielectric constant (ε = 8.4). In this work, we prepared a composite membrane using PVdF-SiO_2_ via phase inversion method. This work was systematically studied towards the morphology, porosity, and electrochemical properties of as prepared membrane. The electrolyte uptake capability of separator membrane tested with 1 M NaPF_6_ electrolyte solution and temperature-dependent ionic conduction test were performed at various temperatures. This membrane exhibits higher ionic conductivity of 4.7 × 10^−2^ S cm^−1^ at room temperature. The physical properties were analyzed by X-ray diffraction, FT-IR, and FE-SEM micrographs analyses. The electrochemical performances with impedance analysis carried for prepared membrane with the as-prepared sodium P2-type cathode material. The material showed an initial discharge capacity of 178 mAh g^−1^ at 0.1 C between 2 and 4 V with 98% columbic efficiency and 81% capacity retention after 50 cycles upon using the as-prepared PVdF-SiO_2_ composite separator membrane.

## 1. Introduction

Worldwide, the research on secondary batteries has gained much interest due to the increasing demand of batteries for powering electronic goods and transport systems (HEV). Lithium batteries are dominating the current market in energy storage area, due to their high power with minimized size, which is desirable for portable electronic applications [1,2,3,4]. As of now, due to increase in prices and limited distribution of lithium around the globe, researchers are on the threshold of searching the alternates [5]. Sodium-ion batteries have gathered attention in the last few decades due to their equivalent electrochemical properties and rich abundance in earth crust that is considered to be the best economic alternative to the Li-ion system [6,7]. However, there are some snags still existing in the sodium-ion battery technology due to their larger ionic radii and sluggish kinetics during the intercalation process, which cause lower performance. Subsequently, there is a need to solve these problems in an alternative ways with innovative simple ideas [8]. Basically, the performance of a battery is based on their electrodes, electrolyte, and ionic conduction properties of the membranes. According to the sequential studies, the performance and life of a battery is limited by the separator membranes and liquid electrolyte. Still, in all types of solid-state battery systems, the parting of electrodes is provided by the ion-conducting polymers, glasses, and porous crystalline ceramics materials. These membranes are present in lithium ion battery prototypes, which have good cyclability, performance, and long life [9]. In contrast, the sodium-ion battery has not thrived to an appreciable level, because the larger Na-ions need separator membranes with highly amorphous natured membrane with larger pore size for free kinetic during the charge/discharge process. Basically, the glass fiber type separators are used for sodium batteries due to their high porosity and good ionic conductivity; however, the leakage of electrolyte makes them unsuitable for practical application where they can cause flammability and explosion of risk associated [10,11]. To overcome these problems, currently the polymer-based separator membranes soaked in liquid electrolytes are suitable for ionic conduction and are leakage-free as safe separator membranes [12,13,14]. The Poly(vinylidene fluoride) (PVdF) is one of the most widely used polymers as membrane materials in the industry for its significant properties like higher thermal and hydrolytic firmness with superior anti oxidation with worthy mechanical forming properties [15]. In addition, the high dielectric constant and strong electron-withdrawing functional groups (C–F) making them much more interesting materials for membrane preparation [16,17]. Many reports are available for PVdF membranes with various preparation techniques, such as surface modification, physical mixing, chemical splicing, etc. [18,19,20]. However, the blending of PVdF with inorganic materials is very interesting because of their suitable operation for forming membranes with good ion conducting nature [19]. Yang et al. [20] reported that poly(vinylidene difluoride-co-hexafluoropropylene) PVdF-HFP-based gel–polymer electrolyte exhibited an ionic conductivity of 0.16 mS cm^−1^ at ambient temperature. Kumar et al. [21] reported PMMA–EC–PC–NaClO_4_-based gel–polymer electrolyte for sodium ion battery with notable ionic conductivity of 3.4 mS cm^−1^ and Harshlata et al. [22] reported polymer electrolyte membrane prepared by phase inversion technique for Sodium ion conduction with ionic conduction value of 0.3 Ms cm^−1^ at room temperature. Janakiraman et al. [23] prepared a porous PVdF-HFP-based separator-cum-gel polymer electrolyte for sodium-ion battery whose ionic conduction value was 1.13 mS cm^−1^.

Herein, an attempt has been made to synthesize high ionic conducting composite membrane using organic/inorganic components. PVdF-SiO_2_ membrane has been prepared via the phase inversion method with various ratios. The major objective of this work is to systematically study the performance of PVdF-SiO_2_ membranes with Na-ion kinetics. The ionic conductivity of prepared membranes has been optimized, and then the optimized membrane has been compared with the commercially available separator membranes, and all other studies were carried out successfully with appropriate interpretation.

## 2. Experimental

### 2.1. Materials

Poly(vinylidene fluoride (PVdF) (C2H2F2)x-) (Sigma–Aldrich, Saint Louis, USA), silicon dioxide nano-powder(SiO_2_-spherical porous) 5-15 nm particle size (Sigma–Aldrich, 99.5%, Saint Louis, USA), *N*-methyl-2-pyrollidone(NMP(C_5_H_9_NO) (Sigma–Aldrich, 99.5%, Saint Louis, USA), sodium hexafluorophosphate (NaPF_6_) (Sigma–Aldrich, 98.0%, Saint Louis, USA), and propylene carbonate (PC) (Sigma–Aldrich,99.7%, Saint Louis, USA) were used without further purification.

### 2.2. Preparation of Porous (PVDF-SiO_2_) Membrane-Phase Inversion Method

The polymer sample of poly(vinylidene fluoride) and silicon dioxide was used for the fabrication of porous membranes by a simple phase inversion process. The various ratios viz, 50:50, 70:30, 80:20, and 90:10 of PVdF and SiO_2_ have been taken to prepare the membranes. The physical status and nature of the prepared films were given in the Supplementary Table S1. It is understood that the flexible nature has been obtained for the low SiO_2_ contents. Beyond 20 wt.% of SiO_2_, the films are turned to be brittle.

Because it was optimized in terms of its physical status and ionic conductivity, the sample contained 90:10 ratio of PVdF and SiO_2_, and exhibited favorable values. Therefore, PVdF and SiO_2_ (90–10 wt.%, 0.9 g PVdF–0.1 g SiO_2_) were taken by dissolving this mixture in 10 mL of NMP solvent by stirring vigorously for 3 h at 60 °C. Transparent white gel was obtained and casted on a glass pate, later it was immersed into deionized water for overnight. Then, the membrane was peeled out from the glass plate automatically. The obtained white sheet membrane was dried under vacuum at 60 °C for overnight to remove the residual solvent before use. The dimension of the obtained membrane was 10 cm × 5 cm (l × b). Finally, the membrane was stored in glove box towards the cell preparation.

### 2.3. Preparation of the P2-Type Na-Fe-Mn-O_2_ Cathode Material

The P2-type Na-Fe-Mn-O_2_ material was synthesized via simple solid-state reaction by using the acetate precursors of the sodium (Na(OOCCH_3_)_2_·4H_2_O (Alfa Aesar, 99.95%, heysham, England), iron (Fe(OOCCH_3_)_2_·4H_2_O (Alfa Aesar, 98%, heysham, England), and manganese (Mn(OOCCH_3_)_2_·4H_2_O (Alfa Aesar, 99%, heysham, England) were used as-purchased. The mixtures are well milled by RETSCH-PM-100 GmbH Planetary type ball miller at 240 rpm for 3 h; the fine mixture was calcined at 950 °C at a heat rate of 5 °C/min in Ar for 14 h; and, finally, the obtained powder placed in a glove box.

### 2.4. Physical Characterization Techniques

X-ray diffraction analysis for pure PVdF, SiO_2_ and prepared samples carried out by using PANalytical X’pertpro diffractometer (Malvern Panalytical, Leyweg, EA Almelo, The Netherlands) with Cu Kα (1.54 Å) radiation in the range 2θ = 10°–70°. A Thermo Scientific Nicolet 380 FT-IR spectrometer was used for analyzing the functional groups in the prepared membrane in the wavenumber range 400 to 4000 cm^−1^. The surface morphology of the porous membrane examined by Scanning Electron Microscope (EVO18 (CARL ZEISS) Jena, Germany). The pore size and thickness of separator membrane estimated using ImageJ software (Laboratory for Optical and Computational Instrumentation, LOCI, University of Wisconsin, Madison, USA). The mechanical stability of membrane was determined using Methact Generic Tensile stress vs. strain measurement system (National Test House (SR), Chennai, India).

### 2.5. Electrochemical Measurements of Polymer Electrolyte Membrane

Sodium hexafluoro phosphate (1 M (NaPF_6_)) solution was prepared with propylene carbonate as solvent. The ability of electrolyte absorption (*EA*) capacity of the membrane of size 20 mm in diameter was calculated by weight before and after dipped into the electrolyte solution for 30 min. Later, the separator membrane was carried out and sponged with filter paper to remove the excess amount of liquid electrolyte. Then, the polymer membrane with absorbed electrolyte was weighed again to calculate the liquid electrolyte acceptance based on the following equation [24].

EA=Aw−Bw÷Bw×100%

where *Bw* and *Aw* are the weight of the membrane before and after dipped into electrolyte solution in (g), respectively. The sodium ionic conductivity (electrochemical impedance spectroscopy (EIS) of the as-prepared separator membrane were analyzed using BCS-815/Electrochemical Analyzer (Bio-Logic, Claix, France) in the frequency range of 10 Hz to 10 kHz. The ionic conduction test with wide range of temperature was performed for the separator membrane, which was sandwiched between stainless steel disks like the electrode with heating mantle set-up in Metrohm Auto lab systems. The ionic conductivity σ (s/cm) calculated of as prepared PVdF-SiO_2_ membrane was calculated using the following.

σ = *l*/AR_b_

Resistance of electrolyte (R_b_) acquired from the intercept of the Nyquist plot with the real axis R_e_(Z), calculated membrane thickness (*l*), and the area of the electrode (A), according to the equation. [25]. The charge–discharge performances of the prepared PVdF-SiO_2_ separator membrane was tested with P2-Na_0.66_Fe_0.5_Mn_0.5_O_2_ material as cathode and sodium metals as anode. Furthermore, electrochemical characterization performed for the P2-Na_0.66_Fe_0.5_Mn_0.5_O_2_ electrode material encompassing the slurry of 85 wt.% active material powder, 10 wt.% conductive carbon black, 5 wt.% PVdF binder, and the NMP used as solvent. The slurry was coated on aluminum foil as current collector and it was dried at 80 °C in vacuum oven for overnight. Then, the CR 2032 coin-type test cell was made with prepared electrode, PVdF-SiO_2_ separator membrane soaked with 1 M NaPF_6_ electrolyte solution. All the preparation of cell couple was carried under inert atmosphere in glove box with moisture level of 0.1 ppm. The galvanostatic charge discharge tests of the cells were conducted at cut-off voltages of 1.8–4.0 V at 0.1 C rate and cyclic voltammetry was performed between 1.5 and 4.5 V at scan rate of 0.1 mV/s.

## 3. Results and Discussion

**Figure 1:**
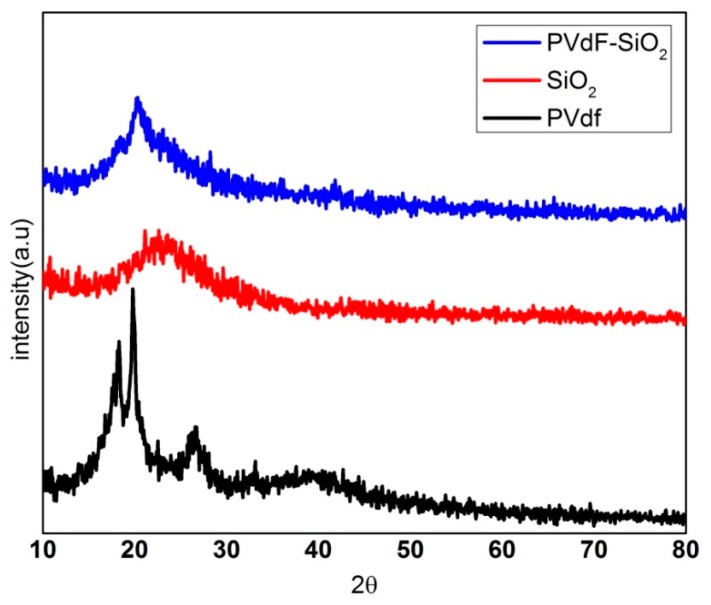
Diffraction pattern of pure PVdF, SiO_2_, and composite of PVdF-SiO_2_, respectively.

Figure 1 shows the X-ray diffraction analysis pattern of the pure PVdF, SiO_2_, and the prepared membrane composed of PVdF-SiO_2_ at 2θ range of 18.3° and 19.8°, 26.6° attributed to the α(010), α(110), and α(021), which can reveal the pure PVdF contains only α-phase [26]. From the diffraction pattern of the PVdF-SiO_2_ separator membrane, it is observed that a strong peak appeared at 2θ = 20.2° assigned to the β-phases viz, β (110) and β (200) planes [27]. It can be clearly concluded that the introduction of SiO_2_ into the pure PVdF changed the α-phase into β phase which elevates the amorphous nature with transformation in the prepared PVdF-SiO_2_ separator membrane. This enhanced the ionic movement for larger Na-ion during the intercalation process.

**Figure 2:**
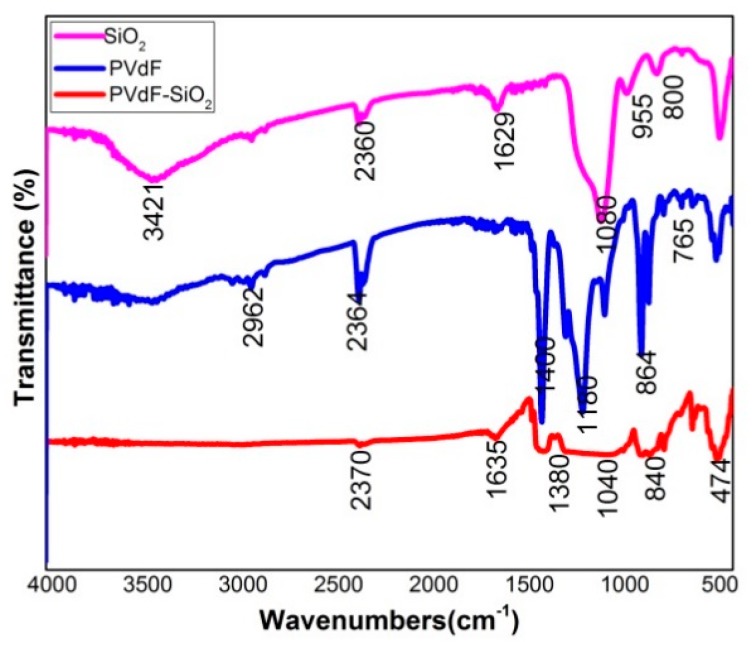
FT-IR spectrum of as prepared PVdF-SiO_2_ membrane, pure PVdF, and SiO_2_.

FT-IR analysis was performed in the range of 400 to 4000 cm^−1^ to confirm the existence of the functional groups present in the PVdF-SiO_2_ porous membrane shown in the Figure 2.

In the FT-IR spectrum, the peaks are observed at the range of 3000 to 3500 cm^−1^ attributed to the –OH stretching, the tiny bending in the wave number around 1635 cm^−1^ assigned to H–O–H bending, the deformation of C–H group indicated at 1380 cm^−1^. The asymmetric stretching of Si–O–Si appeared at 1040 cm^−1^, and the α-Crystal nature of PVdF indicated at 864 cm^−1^. β-phase crystal of PVdF intimated around 474 and 840 cm^−1^ [26,28].

### 3.1. Surface Morphology Analysis

**Figure 3:**
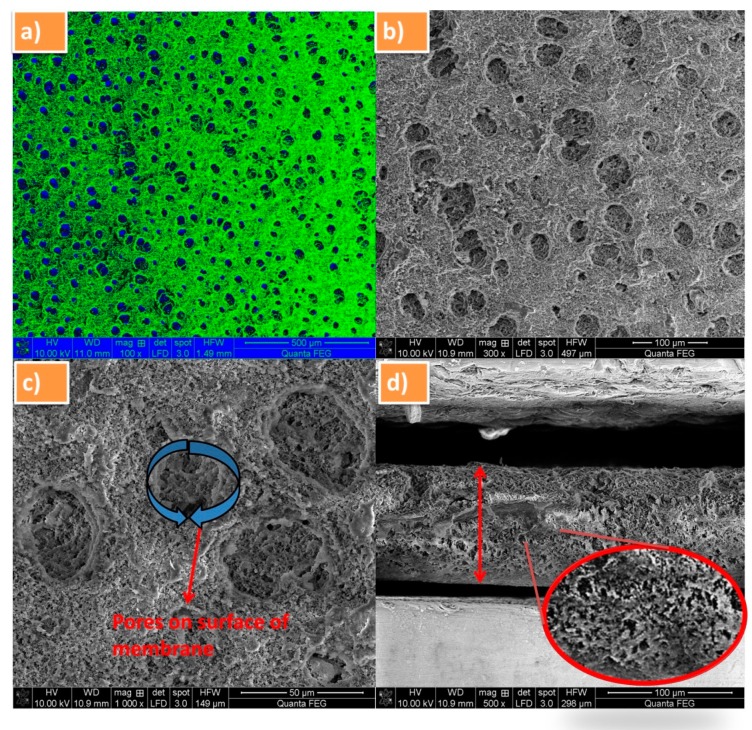
FE-SEM images of PVdF-SiO_2_ membrane (**a**) color overlaid SEM image, the blue and green colors indicate the pores and surface of membrane, respectively; (**b**) separator at 300× magnification; (**c**) visible pores on the membrane surface; and (**d**) cross-sectional FE-SEM view of membrane with enlarged area view.

Figure 3a–d shows the FE-SEM micrographs of the both cross-sectional and surface view of PVdF-SiO_2_ membrane with different magnification. Figure 3a shows the color overlaid image membrane; consequently, the blue and green colors indicate the pores and surface of membrane, respectively. In Figure 3b, pores could be clearly observed in the surface of the membrane at magnification 300×. Figure 3c exhibits the average pore size of 0.7 µm for the prepared PVdF-SiO_2_ membrane. Therefore, this higher porosity of the membrane leads a pathway to greater ionic conductivity in the matrix [29]. The thickness of separator membrane was found as 105 µm that is clearly seen through cross-sectional morphological view (Figure 3d). Moreover, there was no aggregation of SiO_2_ particles existed in the PVdF polymer membrane, evincing that the SiO_2_ particles are evenly dispersed into PVdF matrix during the stirring process.

### 3.2. Electrochemical Analysis

**Figure 4:**
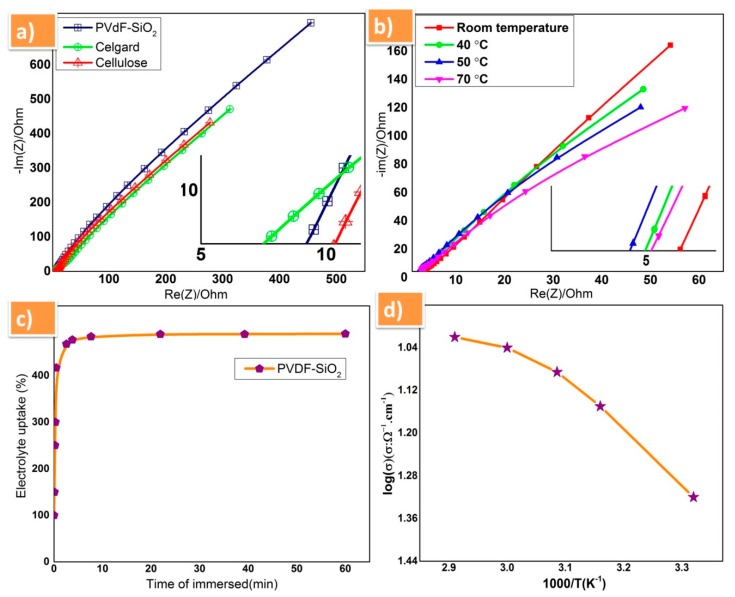
(**a**) Complex impedance spectra of PVdF-SiO_2_, celgard, and cellulose membranes at room temperature; (**b**) ionic conductivity of PVdF-SiO_2_ membrane with different values temperature; (**c**) plot of electrolyte uptake with time; and (**d**) temperature-dependent ionic conductivity plot of PVdF-SiO_2_ membrane.

The ionic conductivity of prepared PVDF-SiO_2_ separator membrane was carried and compared with commercially available celgard-2400 (PP) monolayer membrane and cellulose (NKK, Kochi, Japan) membrane to analyze the performance in sodium electrolyte solution. From the obtained results, the prepared PVdF-SiO_2_ separator membrane showed high ionic conductivity of 4.7 × 10^−2^ S cm^−1^ at room temperature among all samples studied and also higher than previous reports [20,21,22,23]. Furthermore, the ionic conductivity of the prepared PVdF-SiO_2_ was examined at various temperature (room temperature, 40, 50, and 70 °C) it were exhibited good ionic conduction property Figure 4b and the comparison of ionic conductivity of all membrane tabulated in Table 1. The capability of electrolyte uptake of PVdF-SiO_2_ membrane was estimated by soaking in 1 M NaPF_6_ solution for 60 min, and it was plotted in the graph between the time and percentage of liquid electrolyte uptake (Figure 4c); from this graph, one can observe that the membrane has its saturated position within 10 min, which revealed the high porous nature of prepared membrane and also the high amorphous nature of PVdF. The electrochemical performances of PVdF-SiO_2_ composite membrane was analyzed using prepared P2-type Na_0.66_Fe_0.5_Mn_0.5_O_2_ as cathode and sodium metal as an anode and the 1 M NaPF_6_ solution used as electrolyte. The temperature dependent ionic conductivity plot for prepared membrane is shown in Figure 4d. Herein, the change in the linearity of the conductivity plot is attributed to the segmental motion of the polymer comprised in the matrix [30,31,32,33].

**Figure 5:**
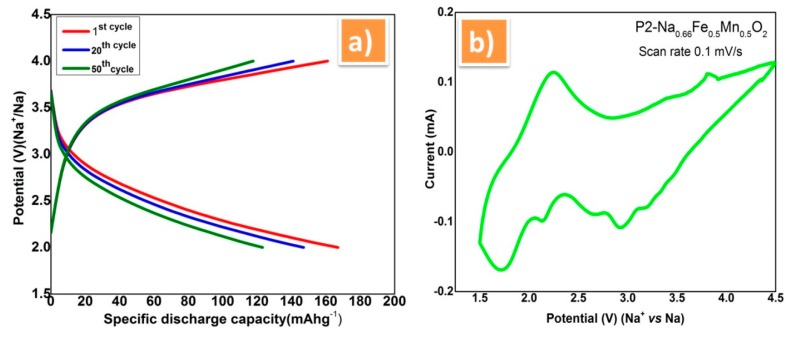
(**a**) Charge–discharge plot of P2-Na_0.66_Fe_0.5_Mn_0.5_O_2_ cathode vs. sodium metal anode using PVdF-SiO_2_ separator membrane between 2 and 4 V at 0.1 C rate. (**b**) Cyclic voltammetry of P2-Na_0.66_Fe_0.5_Mn_0.5_O_2_ using PVdF-SiO_2_ separator membrane.

The typical charge discharge curve of prepared P2-NaFeMnO_2_ material using PVdF-SiO_2_ separator membrane was showed in Figure 5. Here, smooth charge discharge curve were obtained with initial discharge capacity of 178 mAh g^−1^ between 2 and 4 V at 0.1 C rates Figure 5a. Even after 500 cycles, the cell exhibits 98% of columbic efficiency, which were evident for good ionic conduction through the PVdF-SiO_2_ separator membrane. In view of verifying the cycle life of the membrane with the NaFeMnO electrode cell couple, the measurement was extended to 500 cycles. It was found from the cycle life analysis that the discharge voltage of electrode material was stable at 3.7 V without much fading while using this PVdF-SiO_2_ membrane. This shows the ability of the as-prepared PVdF-SiO_2_ membrane is highly harmonious with the sodium electrode and withstanding in more number of cycles. The cyclic voltammetry was performed with the above-mentioned combination of cell couple in the potential range of 1.5 to 4.5 V at the scan rate of 0.1 mV/s, see Figure 5b. In that, all the redox peaks are in good agreement and attributed to the corresponding electrode materials used in the cell according to the previous reports [34,35]. The redox peak at the potential 3.0 V can be attributed to the redox reactions of Fe^2+^/Fe^3+^ during the desertion of Na^+^ ions at the discharging time. The peaks below 2.0 V may be due to Mn redox process [36,37].

The major role of SiO_2_ in PVdF has been envisaged here under.

Herein, the β-phase transformation in PVdF occurs during the incorporation with inorganic fillers (SiO_2_), where it can convince the change in structure due to new nucleation sites in PVdF. That can transform the predominant nonpolar α-phase of PVdF to polar β-phase. Where it may expect to provides free kinetics for Na+ ions in the polymer electrolyte, that can be due to the weaker interaction between the Na+ ion with high electronegative fluorine in the polymer matrix. Basically, in the gel polymer electrolyte type, the ionic liquid introduced in to the polymer network was occupied by the pores in the membrane and then penetrated into the chains of polymer matrix to swell its amorphous domains. As the consequence of these phenomena, the ion carriers can be easily transferred through membrane. Therefore, the increase in ionic conductivity is mostly attributed to the liquid electrolyte where it can be trapped in the pores of the polymer network and the swelling of the amorphous domains by the electrolyte solution [20,38,39,40]. Table 2 corroborates the superior ionic conductivity of the as-prepared PVdF-SiO_2_ composite membrane with previous reports.

## 4. Conclusions

The high ionic conductive polymer PVdF-SiO_2_ composite membranes were synthesized using the phase-inversion method, and the P2-Na_0.66_Fe_0.5_Mn_0.5_O_2_ cathode material was synthesized via conventional solid state methods. The ionic conductivity of the membrane was experimentally verified with various temperature ranges and compared with other type separator membranes. The microstructure, pore size, and thickness of membrane were successfully estimated using software. The electrochemical performance of PVdF-SiO_2_ membrane was verified with P2-Na_0.66_Fe_0.5_Mn_0.5_O_2_ cathode and sodium anode and is higher than the commercial membranes (initial discharge capacity of 178 mAh g^−1^). From the analyses performed in various aspects, one can elucidate that the as-prepared PVdF-SiO_2_ composite membrane could be served as a safe and suitable separator for the upcoming sodium ion battery technologies.

## Figures and Tables

**Table 1:** Ionic conductivity PVdF-SiO_2_ with commercially available membranes.

Separator Type	Electrolyte Solution	Ionic Conductivity(σ) S cm^−1^
Celgard@2400/monolayer (PP)	1 M NaPF_6_/PC	1.6 × 10^−2^ S cm^−1^
Cellulose/NKK Japan	1 M NaPF_6_/PC	1.3 × 10^−2^ S cm^−1^
PVdF-SiO_2_	1 M NaPF_6_/PC	4.7 × 10^−2^ S cm^−1^

**Table 2:** Comparison of ionic conductivity of prepared PVdF-SiO_2_ membrane with previous reports.

S. No.	Type of Separator Membrane	Obtained Ionic Conductivity	Reference
1	PVDF-HEFpoly(vinylidenedifluoride-co-hexafluoropropylene	0.16 × 10^−3^ S cm^−1^	[20]
2	Poly(vinylidene fluoride-hexafluoropropylene)	0.3 × 10^−3^ S cm^−1^	[21]
3	poly(vinylidenefluoridecohexafluororopylene) [P(VdF-co-HFP)]	1.3 × 10^−3^ S cm^−1^	[22]
4	PMMA–EC–PC–NaClO_4_	3.4 × 10^−3^ S cm^−1^	[23]
5	PVdF-SiO_2_	4.7 × 10^−2^ S cm^−1^	This work

## Supplementary Materials

The following are available online at https://www.mdpi.com/2073-4360/12/2/405/s1, Table S1: Physical properties of membrane with different weight ratios of PVdF-SiO_2_.
