# Supplementary material for: Superior Ionic Transferring Polymer with Silicon Dioxide Composite Membrane via Phase Inversion Method Designed for High Performance Sodium-Ion Battery
Source: Polymers (Basel). 2020 Feb 11;12(2):405. doi: 10.3390/polym12020405 (PMC7077619; doi:10.3390/polym12020405)

1. S1. Physical properties of memrane with different weight ratios of PVdF-SiO_2

S.no	PVdF-SiO_2 (Weight %)	Physical appearance of memrane	Flexiablity of memrane	Digital image of membrane
**1**	50-50	Milk white in color/rigid	More brittle	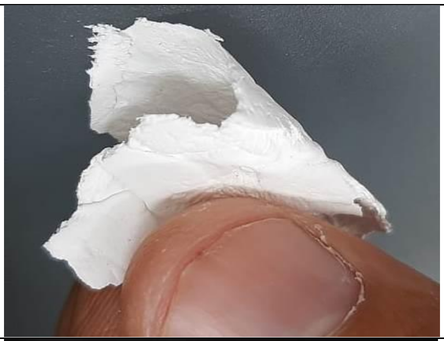
**2**	70-30	Milk white in color/rigid	brittle	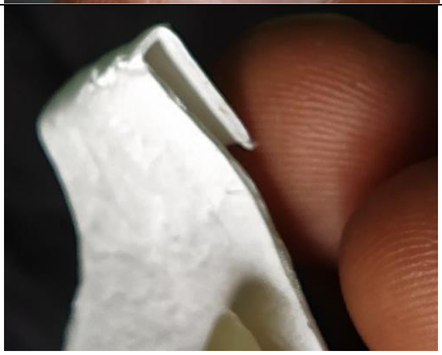
**3**	80-20	Milk white in color/soft	flexible	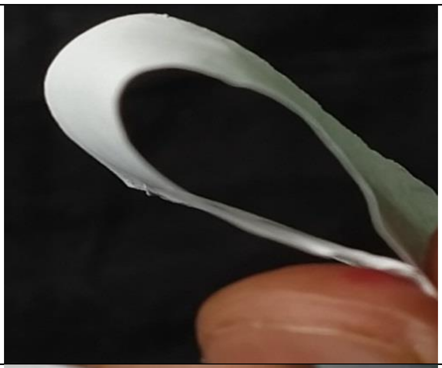
**4**	90-10	Milk white in color/soft	More flexible	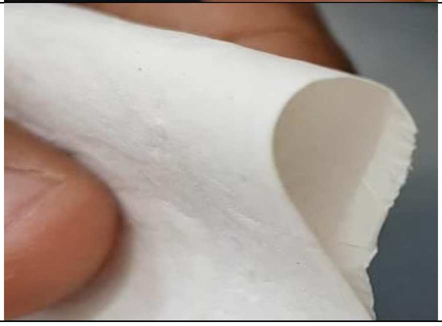

2. ST1 Comparison of separator thickens with commercial thickness.

S.No.	Type of separator	Thickness (μm)
1	Celgard Polypropalyne 2400 (PP) Monolayer	50
2	NKK cellulose(TF40)	35
3	PVdF-SiO_2	90-110

3. Mechanical stability of as prepared PVdF-SiO_2 membrane with electrolyte solution

The mechanical stability of electrolyte membrane socked in electrolyte solution was determined using Methact Generic Tensile-stress *vs* strain measurement system, (from National Test Houseversion 10.2.0.0), and the stress value of 2.33 MPa with moderate elongation of 9.81% was observed.

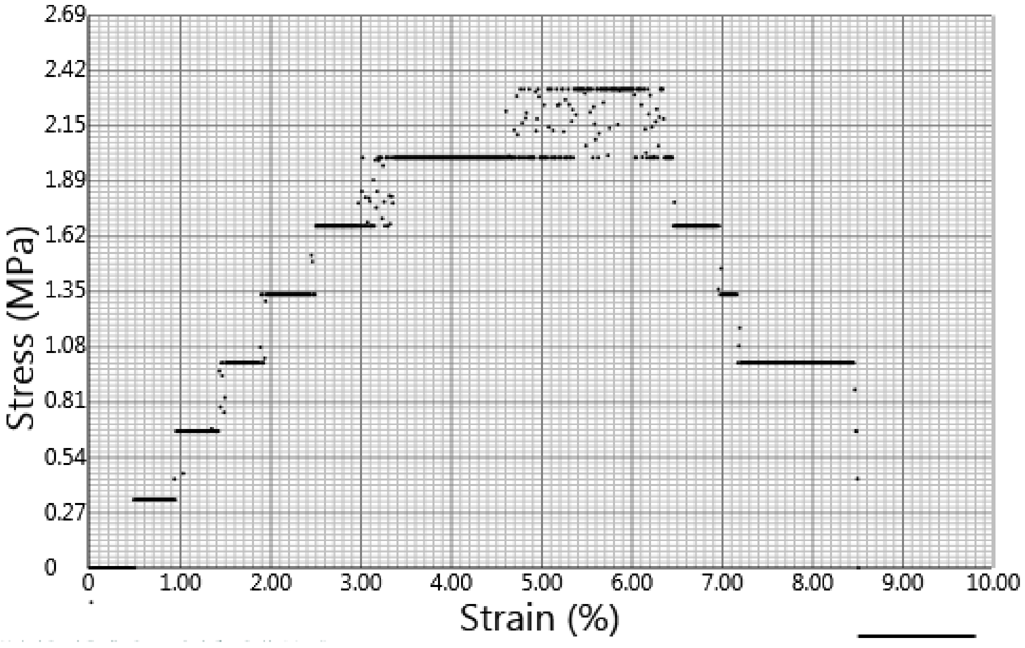

4. Mechanical stability of membrane different ratios PVdF-SiO_2.

The mechanical stability of prepared membrane was obtained for samples of ratio (a) 90:10%, (b) 80:20% PVdF-SiO_2 contents respectively .The membrane of PVdF-SiO_2 90:10% shows high mechanical stability by means of higher stress value around 4.67 MPa with total elongation around 9.41% . The sample of 80:20% PVdF-SiO_2 ratio shows low stress value 3.33 MPa with elongation of 7.42% when compare to 90:10% PVdF-SiO_2 membrane. Form the observation the when reach the certain amount of SiO_2 content in the PVdF polymer matrix reduce mechanical

stability of membrane due to the steric effect. The membrane with 70:30, 50:50% of PVdF-SiO_2 are brittle so the samples are not suitable for experiment.

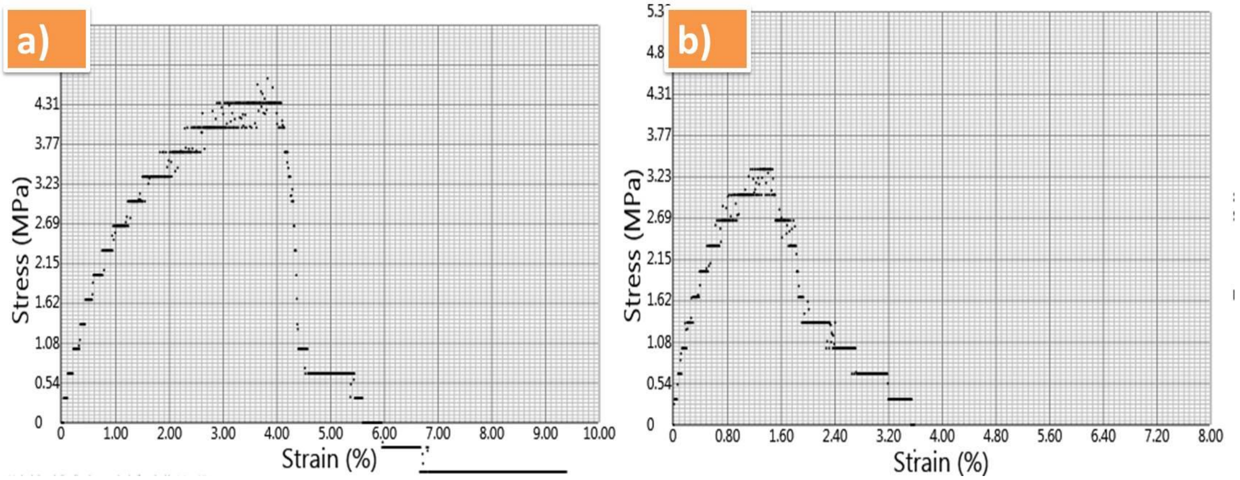

Supplement: Supplementary file 1 [file polymers-12-00405-s001.pdf]
